# Urinary schistosomiasis among vulnerable children in a rehabilitation home in Ibadan, Oyo state, Nigeria

**DOI:** 10.1186/s12879-017-2591-6

**Published:** 2017-07-11

**Authors:** Obioma Uchendu, Victoria Oladoyin, Michael Idowu, Oluwapelumi Adeyera, Oluwatosin Olabisi, Oluwafisayomi Oluwatosin, Gbemisola Leigh

**Affiliations:** 1 Department of Community Medicine, College of Medicine, University of Ibadan, Ibadan, Nigeria; 2 Department of Community Medicine, University College Hospital, Ibadan, Nigeria

**Keywords:** Schistosomiasis, Migrant worker, Child health

## Abstract

**Background:**

Schistosomiasis is a disease of public health importance with long term complications mostly common among children, rural dwellers, poor and migrant workers. Studies have not documented the burden among migrant workers and their families. The study aimed to describe the burden of schistosomiasis and demographic characteristics among children of migrant workers residing in a rehabilitation home in Ibadan, Nigeria.

**Methods:**

A cross-sectional study using sixty six children, who were tested following complaints of haematuria by six of them. An interviewer-administered questionnaire was used to collect information on demographic and environmental characteristics of the children and urine microscopy, was conducted. Data was analysed using descriptive statistics and correlation. Statistical significance was set at 5%.

**Results:**

Mean age of respondents was 11.8 ± 4.0 years and 57.6% were males. The prevalence of schistosomiasis was 19.7% with preponderance among males (64.3%) and children aged 12 years and above (71.4%); 85.7% of infected children were from Kwara State; 78.6% waded in water body and 92.9% had red blood cells and pus cells on urine microscopy.

**Conclusions:**

The burden of schistosomiasis is high among children of migrant workers and they serve as reservoirs for transmission of the disease. Government needs to work synergistically with NGOs, FBOs and other partners to achieve schistosomiasis prevention and control among this particular group.

## Background

Schistosomiasis is one of the Neglected Tropical Diseases (NTDs) caused by parasitic worms of the genus Schistosoma. Schistosomiasis is an acute and chronic disease which is endemic in about 78 countries. In 2014, about 258 million people required preventive treatment for schistosomiasis, out of which 90% were from Sub-Saharan Africa [1]. Schistosomiasis is the second most common and overwhelming parasitic disease after malaria which accounted for more than 200,000 deaths in Sub-Saharan Africa [2].

Nigeria has the highest burden of schistosomiasis in the world. The overall prevalence was 9.5% and it was found present in all the 20 states surveyed but was higher in the northern part of Nigeria [3]. More than two-third of those affected by the disease required intervention for schistosomiasis. Furthermore, a study conducted in Anambra State reported a prevalence of 15.7% [4] while another in Maiduguri reported a prevalence of 14.5% [5].

Schistosomiasis is also the most prevalent waterborne disease and is considered the biggest risk to rural dwellers in places with inland water in developing countries [6]. The disease is an important occupational hazard for water-dependent professions like agriculture and fish farming [7, 8]. School aged children who live in areas with water bodies that habour the vectors are most susceptible to the diseases. This is because they go to the water bodies for domestic, recreational, farming, fishing and social activities where they become exposed to the parasite [3, 8].

Schistosomiasis is a major public health problem in Nigeria but has received little attention. Reasons for the significant burden of the disease includes: poverty, poor environmental sanitation and water supply and poor access to health care facilities. These communities are also hard to reach and therefore underserved by policies and programs aimed at controlling schistosomiasis and other NTDs [9]. There has been no sustained decrease in the prevalence of disease as the prevention, control and treatment programs in Nigeria has been sub-optimal [10]. In 2013, more than 60 million people required treatment for schistosomiasis, however, treatment programs were available for only few of the affected population [11]. The Carter Center along with other development and implementation partners work with the Federal Ministry of Health (FMOH) in Nigeria to treat school aged children in priority states for the disease; however these control programmes are yet to have a noticeable effect on the prevalence of schistosomiasis therefore raising doubts if control can be achieved [12].

Migration by man which traditionally involves movement from one geographical location to another for social, economic, political or financial reasons now involves his movement of disease causing organisms. Work-related migration occurs as a result of social unrest, better land, lack of infrastructure, low wages, crisis and war, poor climatic conditions and unfavorable land especially by farmers [13]. Data on internal migration are unavailable in most developing nations due to poor systematic data collation and lack of internal boundaries coordination in these countries [14]. Migrant workers are an important and peculiar group of people because they are prone to different social and health problems and contribute to disease burden [15]. They include farmers, miners, transporters, commercial sex workers. Migrant farmers often move with their families to new places and as such, the health and education of children from such families are compromised either due to poor standards or total lack of health and educational services [15]. These children have no access to vaccination from vaccine preventable diseases and quality education. Subsequently they are exposed to prevalent communicable diseases where they live including the neglected tropical diseases, malnutrition and child labour [16].

Migration has been listed as an important factor influencing the prevalence of schistosomiasis therefore making migrant populations a priority group in the control of schistosomiasis. When an infected person migrates, the disease is introduced into the new area thereby creating new risk zones [17]. One of the recommended actions for schistosomiasis control by World Health Organisation (WHO) is to improve the health of the migrant population. This recommendation is ranked as the third most important control strategy further stressing the peculiarity and importance of migrant workers [17]. Since the contribution of government in the control of schistosomiasis has been inefficient largely due to poor access to these vulnerable population groups (migrant workers) who live in hard to reach areas, there is a need to explore other ways to increase access to health and educational services to them. In several hard to reach communities, health, educational and social interventions are provided by Non-Governmental Organizations (NGOs) and Faith-Based Organizations (FBOs).

This study documented the burden of schistosomiasis among the vulnerable children who reside in a rehabilitation centre.

## Methods

Oyo State is one of the 36 states in Nigeria. It is bounded in the south by Ogun State and in the North by Kwara State, in the west it is bounded partly by Ogun State. It was created in 1976 out of the old Western Region and has an estimated population of 5,591,589 according to the 2006 Provisional Census figures released by the National Population Commission. Oyo State is an inland state in south-western Nigeria, with its capital at Ibadan. Ibadan has a population of about 1.4 million according to the 2006 census and is divided into 11 local government areas (LGAs) each of which is further divided into wards [18].

**Fig. 1:**
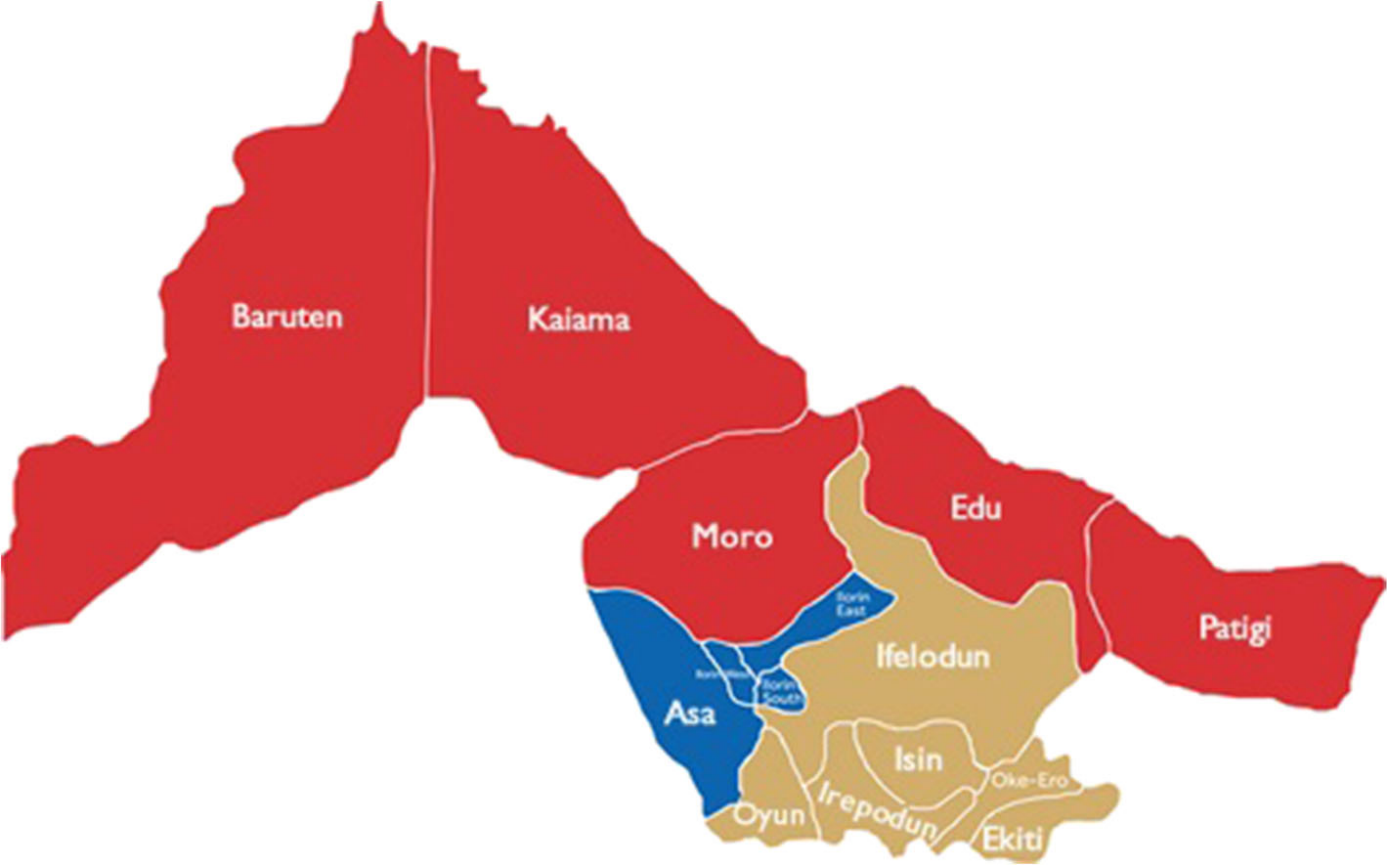
Map of Baruten Local Government Area

This study was conducted in a rehabilitation center for orphans and vulnerable children coordinated by Living Word Mission (aka La Vie Mot) in Ibadan, Oyo State, Nigeria. Living Word Mission (LIWOM) is a nonprofit, Faith Based Organization (FBO) working in rural and urban areas of four states including Oyo State of the South Western part of Nigeria. The organization was established in 1996 and registered as an FBO/NPO (Non-Profit Organisation) in 2010. The organization provides shelter, educational, health care, nutritional, legal protection and psychosocial support to Orphans and Vulnerable Children (OVCs) and young adults. Sixty six (66) vulnerable children were resident in the facility at the time of the study. Some of the children were from migrant families who were brought to the facility because of their severe vulnerability following parental consents. These children however return to their families during school holidays. The children in this center come from different states across the country, one of which is Kwara State. This state in North Central Nigeria has been categorized as one of the states with moderate risk of infection (between 10% and 49%) in Nigeria [19]. Baruten as one of the Local Government Areas (LGA) in Kwara State is a predominantly rural LGA with poor infrastructural facilities (Fig. 1). Economic activities in this LGA include, farming, fishing, hunting and cattle rearing [20]. The Rehabilitation and Social Medicine Unit, Department of Community Medicine in University of Ibadan works with several organizations that provide rehabilitative services for vulnerable population groups. The Unit provides technical and medical support such as training of the staff, needs and environmental assessments, health education, screening (including the staff) and treatment of common diseases. LIWOM is one of the facilities the unit works with where proper evaluation following complaints of haematuria by six of the resident children was conducted for all the children.

An interviewer-administered, semi-structured questionnaire adapted from the schistosomiasis manual [21] was used to obtain information on their socio-demographic and environmental characteristics, history of wading in water and history of haematuria. Urine samples were also obtained from the children for macroscopic and microscopic analysis. All procedures were performed in duplicates for quality assurance [22] by a medical laboratory scientist. Data was gotten in May, 2014 and all 66 children residing in the home were tested.

Urine microscopy: For each child, 15 ml of mid-stream urine specimen was collected into a clean dry universal bottle and was examined macroscopically. Two drops of saponin was then added to samples that had visible blood. The procedure for preparing urine specimen to examine Schistosome eggs was then performed [23]. Using the microscope under 10× and 40× objective lens, Pus cells, RBC cells, epithelial cells, yeast cells and crystals were observed for and counted where present. Also, the number of Schistosoma eggs were counted and reported in number of egg per 10 ml of urine.

Data was entered using IBM-SPSS version 20 (International Business Machine-Statistical Package for Social Sciences) and analyzed using descriptive statistics. Frequencies were reported for the socio-demographics and urinalysis. Bivariate analysis was conducted and statistical significance was set at 5%.

For Urine microscopy, pus cells and Red Blood Cells (RBC) < 5 cells/hpf were considered normal; Pus cells, RBC cells, epithelial cells, bacteria, crystals and yeast in the urine samples were then reported as frequencies. Schistosoma haematobium egg counts was categorized into: light (1–9 eggs/10 ml), moderate (10–49 eggs/10 ml) and heavy (>50 eggs/10 ml) infection.

Ethical approval was obtained from the Oyo State Ministry of Health and consent was gotten from the guardians in the home. The study was conducted in accordance to the Declaration of Helsinki. The cases were reported to the Medical Officer of Health for Ibadan South West Local Government Area, where the home was located and drugs were provided for the treatment of infected children in accordance with national guidelines for reporting and treatment of NTDs.

## Results

### Socio-demographics

The mean age of respondents was 11.8 ± 4.0 years with 57.6% being males. Almost half of the respondents (45.5%) were from Kwara State while about one-third (30.3%) were from Oyo State. About two-fifth (40.9%) of the respondent’s fathers were farmers. (Table 1). The respondents from Kwara State (45.5%) were the migrant population.

**Table 1:** Sociodemographic and environmental characteristics of residents of LIWOM rehabilitation centre, Ibadan, Nigeria, 2014

Variable (*N* = 66)	Confirmed schistosomiasis n (%)		Total	Chi-square	*p*-value
	Yes (*n* = 14)	No (*n* = 52)			
Age					
Less than 12 years	4 (28.6)	24 (46.2)	28 (42.4)	1.396	0.362
12 years and above	10 (71.4)	28 (53.8)	38 (57.6)		
Sex					
Male	9 (64.3)	29 (55.8)	38 (57.6)	0.328	0.762
Female	5 (35.7)	23 (44.2)	28 (42.4)		
Source Population					
Kwara State^c^	12 (85.7)	18 (34.6)	30 (45.5)	11.616	0.001*
Other states^a^	2 (14.3)	34 (65.4)	36 (54.5)		
Father’s Occupation					
Farming	7 (50.0)	20 (38.4)	27 (40.9)	0.607	0.544
Others^b^	7 (50.0)	32 (61.6)	39 (59.1)		
Mother’s Occupation					
Farming	7 (50.0)	17 (32.7)	24 (36.4)	1.310	0.252
Others^b^	7 (50.0)	35 (67.3)	42 (63.6)		
Presence of water body					
Yes	12 (85.7)	31 (59.6)	43 (65.2)	3.309	0.113
No	2 (14.3)	21 (40.4)	23 (34.8)		
History of Wading					
Yes	11 (78.6)	26 (50.0)	37 (56.1)	3.655	0.073
No	3 (21.4)	26 (50.0)	29 (43.9)		

^a^Nassarawa, Bauchi, Kebbi, Kogi, Plateau. Edo, Oyo, Anambra

^b^Missionary, trader, driver, bricklayer, printer, housewife

^c^Kwara state = migrant population

^*^Significant association

### Prevalence of schistosomiasis

**Fig. 2:**
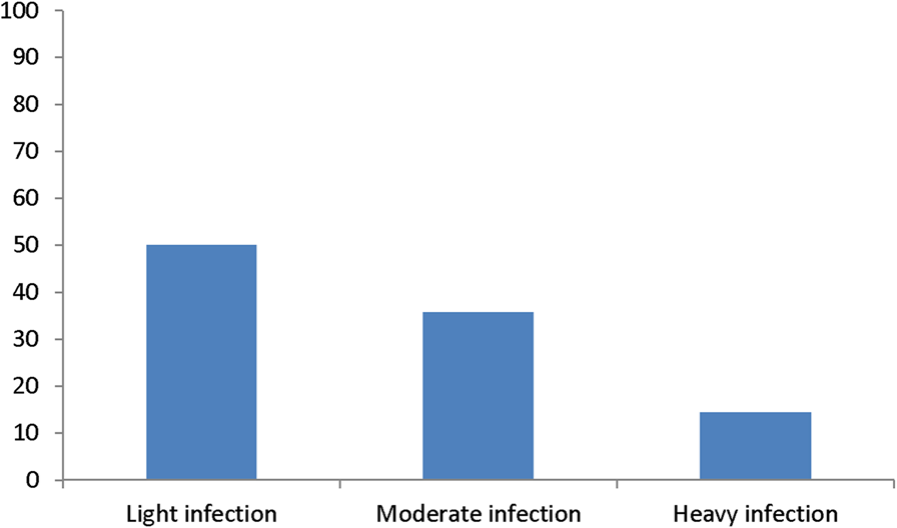
Intensity of Schistosomiasis Infection

The prevalence of schistosomiasis was 21.2% with preponderance among males. Among children infected with schistosomiasis, there were 64.3% males and 71.4% aged 12 years and above. Over three-quarter (78.6%) of infected children waded in water body. And 92.9% had microscopic haematuria with a median egg count of 9.5/10 ml (2.0–172.0). Regarding the intensity of infection, 7 (50%) had light infection; 5 (35.7%) had a moderate infection while 2 (14.3%) had a heavy infection (Fig. 2).

### Urine microscopy

The urine microscopy indicated that children with schistosomiasis had more pus cells (92.9%) and RBC cells (92.9%) in their urine (Table 2).

**Table 2:** Urine microscopy results of residents of LIWOM rehabilitation centre, Ibadan, Nigeria, 2014

Variables (*N* = 66)	Confirmed schistosomiasis n (%)		Total
	Yes (*n* = 14)	No (*n* = 52)	
Pus cells			
0–4/hpf	1 (7.1)	40 (76.9)	41 (62.1)
> 4/hpf	13 (92.9)	12 (23.1)	25 (37.9)
RBC cells			
0–4/hpf	1 (7.1)	51 (98.1)	52 (78.8)
> 4/hpf	13 (92.9)	1 (1.9)	14 (21.2)
Epithelial cells			
Yes	14 (100.0)	49 (94.2)	63 (95.5)
No	0	3 (5.8)	3 (4.5)
Yeast cells			
Yes	1 (7.1)	1 (1.9)	2 (3.0)
No	13 (92.9)	51 (98.1)	64 (97.0)
Bacteria cells			
Yes	14 (100.0)	27 (51.9)	41 (62.1)
No	0	25 (48.1)	25 (37.9)

## Discussion

Neglected Tropical Diseases (NTDs) are a group of parasitic and bacterial infections that are found in tropical and subtropical areas like the Sub Saharan Africa [2]. This study documented the burden of schistosomiasis among children living in a rehabilitation home for vulnerable children where 30 of the 66 children were from Baruten, Kwara State. The prevalence of schistosomiasis in this study was 21.2%. Of the 14 infected children, 12 (85.7%) were from Baruten, therefore, 40% of children in this study from Baruten were infected with schistosomiasis. Baruten Local Government Area has approximately 74,981 children aged 5–19 years old [18]. Extrapolating the 40% prevalence from this study to the population of children in Baruten, would approximate that 29,992 children are infected with schistosomiasis there-in. Studies in Anambra and Maiduguri reported lower prevalence (15.7% and 14.5% respectively), whereas the national prevalence from a 2013 survey reported a prevalence of 9.5% [3–5]. The study population was vulnerable children living in a rehabilitation home compared to the general school-aged children in other studies accounting for the difference in prevalence.

The higher preponderance among males and older children from this study has been reported consistently in various studies across Nigeria [3, 24–27]. The risk factor for this preponderance is frequency and duration of exposure to water bodies by children where the snail which is the intermediate host for S. haematobium resides in water bodies and sheds the Schistosome cercariae [8, 26, 28]. Males tend to have more contact with water bodies especially for recreational purposes especially during day-time when eggs are been shed while the females’ exposure is mostly limited to domestic chores (laundry, fetching) [8, 26]. Also, poor access to education for children of migrant workers reduces time spent in school and increases exposure time to water bodies. Schistosomiasis intensity reports have also indicated that most infected children have light to moderate infection similar to this study [3]. Furthermore, the disease is associated with non-fatal morbidity like mild anaemia, urinary tract infection and malnutrition (short-term) [8]; but chronic and untreated schistosomiasis may lead to serious morbidities like organ failure, and increased risk of bladder cancer or even death (long-term) [6, 27].

Some of the children from LIWOM have migratory workers as parents and required educational and health interventions. Schistosomiasis is a disease of poverty affecting people living in rural areas, have poor access to health facilities and are migratory workers [6, 26, 29]. This migratory population is important in the control of schistosomiasis. As they move from one place to another they serve as reservoirs of transmission for the disease, therefore infecting new people [17]. The children from LIWOM go back home for holidays and may be possibly re-infected after treatment. These children can serve as change agents when they go back home, informing other children and adults of the dangers of wading through water bodies and educating them on schistosomiasis infection. Even though there are no figures for prevalence of schistosomiasis among the migrant population [17], they are a peculiar group that should be studied. Existing interventions seldom benefit hard-to-reach populations where NGOs and FBOs work to provide social services. There is a need for a synergistic approach between the government, partners and these NGOs in the hard-to-reach areas to reduce the prevalence of this disease. Without this, the burden of schistosomiasis will continually persist.

The prevalence obtained from this study may have been overestimated because the children were from the same area which does not give a true representation of the burden of the disease in Baruten. However, the children from Baruten were not brought to the rehabilitation home based on the history of haematuria and so the prevalence obtained may still be a reflection of the burden. Another limitation of the study is the small study population, however, this does not nullify the results observed considering their source population. It is therefore recommended that a community-based should be conducted to determine the true prevalence of schistosomiasis in the Baruten LGA. There is also a need for synergism between the community members, local health authorities, NGOS/CBOS towards providing prevention, control and treatment intervention in the LGA.

## Conclusion

The burden of schistosomiasis among children from Baruten LGA is high. The consequences of this infection among this vulnerable group include haematuria anaemia, and features of urinary tract infection. Health education and mass chemotherapy involving NGOs and FBOs in such hard-to-reach population should be provided to control the burden of schistosomiasis in Baruten and other hard-to-reach areas.

## Abbreviation

FBO: Faith-Based Organisation
FMOH: Federal Ministry of Health
LIWOM: Living Word Mission
NGO: Non-Governmental Organisation
NTD: Neglected Tropical Disease
OVC: Orphans and Vulnerable Children
RBC: Red Blood Cells
WHO: World Health Organisation
